# Allogeneic limbo-deep anterior lamellar keratoplasty (Limbo-DALK)—A novel surgical technique in corneal stromal disease and limbal stem cell deficiency

**DOI:** 10.1371/journal.pone.0298241

**Published:** 2024-02-12

**Authors:** Verena Schöneberger, Volkan Tahmaz, Mario Matthaei, Sigrid Roters, Simona L. Schlereth, Friederike Schaub, Claus Cursiefen, Björn O. Bachmann

**Affiliations:** 1 Department of Ophthalmology, Faculty of Medicine and University Hospital Cologne, University of Cologne, Cologne, Germany; 2 Department of Ophthalmology, University Medical Centre Rostock, Rostock, Germany; All India Institute of Medical Science - Bhopal, INDIA

## Abstract

**Purpose:**

To describe a novel corneal surgical technique combining Deep Anterior Lamellar Keratoplasty (DALK) with grafting of allogeneic limbus (Limbo-DALK) for the treatment of eyes with corneal stromal pathology and limbal stem cell deficiency (LSCD).

**Methods:**

Clinical records of six Limbo-DALKs performed in five patients diagnosed with LSCD and corneal stromal pathology requiring keratoplasty were retrospectively reviewed. All patients were diagnosed with LSCD due to various pathologies including thermal and chemical burns, congenital aniridia or chronic inflammatory ocular surface disease. Parameters analysed included demographics, diagnoses, clinical history, thickness measurements using anterior segment OCT, visual acuity, and epithelial status. Regular follow-up visits were scheduled at 6 weeks as well as 3, 6, 9, and 12 and 18 months postoperatively. Main outcome measures were time to graft epithelialisation and the occurrence of corneal endothelial decompensation.

**Results:**

Two grafts showed complete epithelial closure at 2 days, two at 14 days. In one eye, complete epithelial closure was not achieved after the first Limbo-DALK, but was achieved one month after the second Limbo-DALK. No endothelial decompensation occurred except in one patient with silicone oil associated keratopathy. Endothelial graft rejection was not observed in any of the grafts.

**Conclusion:**

Based on the data from this pilot series, limbo-DALK appears to be a viable surgical approach for eyes with severe LSCD and corneal stromal pathology, suitable for emergency situations (e.g. corneal ulceration with impending corneal perforation), while minimising the risk of corneal endothelial decompensation.

## Introduction

The healthy corneal epithelium is constantly renewed by cells derived from limbal corneal epithelial stem cells [1]. Several pathological conditions can harm these cells, resulting in limbal stem cell deficiency (LSCD), in which fibrovascular conjunctival epithelium replaces the corneal epithelial cells. A small proportion of limbal stem cells may be sufficient to epithelialise the entire cornea [2]. LSCD can occur in congenital aniridia, post-traumatic after thermal or chemical damage to the limbus, after repeated ocular surgery, or in association with chronic inflammatory diseases of the ocular surface. LSCD frequently results in functional blindness in the affected eye [3]. In addition to surface opacity and recurrent epithelial defects, these patients often have recurrent stromal ulceration with stromal scarring, thinning or corneal perforation [4, 5].

If conventional penetrating keratoplasty is performed in eyes with combined stromal pathology and LSCD, conjunctivalisation of the initially clear graft, corneal endothelial rejection and subsequent graft failure are very likely. In LSCD, limbal stem cells or alternative epithelial stem cells must be grafted to ensure sufficient epithelial coverage of the corneal surface. Allogeneic penetrating central limbo-keratoplasty uses decentralised trephination of the donor cornea to create a graft that contains additional limbal tissue for several clock hours [6–8]. This method was first described in 1996 by Sundmacher and Reinhard et al. and further developed in the following years. [6–8] The donor tissue with a crescent-shaped limbal area of up to 40% of the circumference is implanted centrally into the recipient’s cornea [1, 6, 7]. Following such transplantations, rejections often occurs not only against the grafted limbal stem cells but also against the allogeneic corneal endothelium, even with systemic immunosuppressive treatment is performed [1, 9].

Over the past 20 years, the number of lamellar corneal transplants has increased steadily in many regions for a variety of indications [10]. Lamellar procedures such as Descemet stripping automated endothelial keratoplasty (DSAEK), Descemet membrane endothelial keratoplasty (DMEK) or Deep Anterior Lamellar Keratoplasty (DALK) offer advantages over penetrating keratoplasty (PK) in cases where the recipient’s remaining corneal layer is healthy and unaffected by the corneal disease. The advantage of DALK over PK is a higher postoperative endothelial cell count and a lower risk of endothelial decompensation due to the absence of endothelial rejection [11–13].

One case report describes deep anterior lamellar limbo-keratoplasty for bilateral limbal stem cell deficiency in two cases. However, a graft of 11 to 11.5 mm diameter was used, which included the entire cornea including the limbus [14]. We postulate an alternative, even more tissue sparing approach in which, a normal graft diameter is chosen, analogous to the limbo keratoplasty technique, while preserving the patient’s own endothelium. This ensures a distance to the usually more vascularised sclera and conjunctiva during the initial phase of wound healing, which is accompanied by increased inflammatory activity. Moreover, this approach allows for a safer dissection in emergency situations with impending corneal perforation, where extensive large diameter corneal dissection increases the risk of perforation of Descemet’s membrane.

We propose a low postoperative rate of endothelial decompensation when limbo-keratoplasty is performed while preserving the healthy endothelial cells of the recipient. We named this new surgical procedure Limbo-Deep Anterior Lamellar Keratoplasty (Limbo-DALK). Here we describe the surgical technique and first results of Limbo-DALK.

## Methods

In this retrospective study, we reviewed clinical records of consecutive Limbo-DALKs performed for LSCD and corneal stromal pathology between January 2020 and April 2022 at the Department of Ophthalmology, University of Cologne, Cologne, Germany. Local ethics committee approval has been obtained for retrospective evaluation (15–301). All tenets of the Declaration of Helsinki were adhered to. Authors had access to the clinical records of the included patients during and after data collection.

### Inclusion and exclusion criteria

All Limbo-DALK surgeries performed in our clinic between January 2020 and April 2022 were reviewed and a total of 6 patients were identified. We excluded one patient who had undergone penetrating keratoplasty prior to Limbo-DALK. One patient received two consecutive Limbo-DALKs on the same eye, which were treated as separate cases.

### Donor preparation

**Fig 1:**
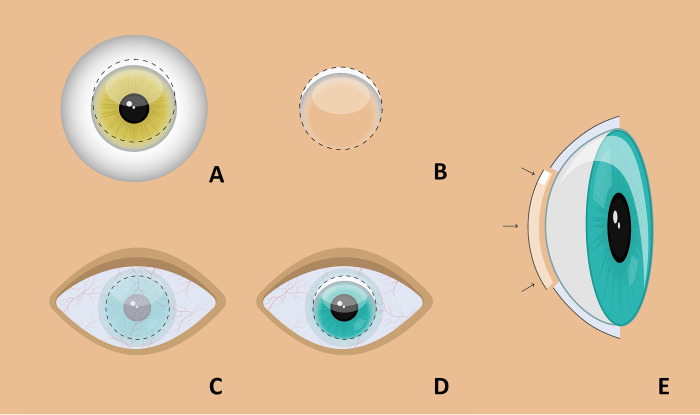
Limbo-DALK preparation and transplantation. **A, B** Donor preparation for Limbo-DALK. After removal of Descemet’s Membrane, an upward decentration trephination (A; dotted line) results in a graft comprising up to 4 clock hours of donor limbus. **C-E** Limbo-DALK surgery in the recipient. A circular incomplete trephination to 90% of the corneal stromal thickness was performed in the centre of the recipient’s cornea **(C)**. The bottom of the trephination served as the starting point for lamellar preparation. After removal of the stroma, the donor lamella with the limbus was placed centrally in the recipient bed **(D)**. This technique preserved Descemet’s membrane and the patient’s corneal endothelium **(E)**.

Corneal grafts suitable for tissue donation without stromal or limbal abnormalities were used for Limbo-DALK. In two patients for whom surgery could be planned, an HLA-typed graft was used. During donor preparation, the donor tissue was placed endothelium up on a punch block and Descemet’s membrane (DM) was removed after staining with trypan blue. The donor cornea was then trephined decenterally from the endothelial side resulting in a graft comprising up to 40% of the superior limbus (Fig 1A and 1B).

### Limbo-DALK surgery

After central marking of the cornea with a claw, circular incomplete trephination of the peripheral cornea was performed using a Hessburg-Barron vacuum trephine (Barron Precision Instruments, Grand Blanc, Michigan, USA) to approximately 90% of the minimum corneal stromal thickness (Fig 1C–1E). In all cases manual dissection of the corneal stroma as close as possible to the level of DM was performed as previously described [15]. The anterior donor lamella was fixed with 10–0 nylon single sutures with the attached limbus oriented either in the 12 o’clock direction or in the direction of the highest degree of LSCD. The knots of the sutures were countersunk. To achieve an even surface level, gross incongruences in donor and recipient thickness at the interface were corrected by adjusting the suture depth and tension. In one patient the thickness of the peripheral donor cornea was reduced with scissors (both surgeries patient no. 1). In two cases sulphur hexafluoride 20% (SF6 20%) was used to add pressure to the recipient endothelium and DM to the donor tissue in the presence of DM defects. In addition, Bevacizumab was injected subconjunctivally in four out of six patients and a bandage contact lens was applied in one patient.

### Postoperative therapy

Postoperative medication included topical prednisolone acetate 1% or dexamethasone 1% five times daily in tapering dosage over at least 12 months and unpreserved topical antibiotic eye drops with ofloxacin for two weeks, as well as preservative-free artificial tears (five times a daily). In addition, in the two cases with gas in the anterior chamber Pilocarpine 2% eye drops were applied three times a day for as long as the gas covered the lower edge of the pupil, and the patients were instructed to maintain a strict supine postoperative position for at least for three days, with only bathroom privileges and continuous intraocular pressure (IOP) monitoring. ^20^

Due to the high-risk constellation in these cases, systemic immunosuppression was prescribed, initially with cortisone and mycophenolate mofetil 1 gram twice daily, reduced to mycophenolate mofetil alone after 4 weeks. Immunosuppressive therapy was maintained for at least 1 year. One case with herpetic corneal disease received no immunosuppressive therapy, but long-term topical Ganciclovir and systemic Acyclovir and Prednisolone.

### Postoperative follow-up

Follow-up examinations were scheduled to revisit patients at 6 weeks and 3, 6, 9, 12 and 18 months postoperatively with final suture removal at least 18 months after surgery. Primary outcome was corneal graft survival. Postoperative significant graft detachments requiring re-injection of gas into the anterior chamber (re-bubbling with SF6 20%) to reattach the donor tissue to the Limbo-DALK graft as well as removal of loose sutures or graft dehiscence requiring re-suturing were documented.

We analysed the following slit-lamp findings: epithelialisation and vascularisation of the cornea, vascularisation of the recipient and, if applicable, of the donor tissue, as well as the occurrence of endothelial decompensation and immunological graft reactions. Endothelial decompensation was defined as central loss of graft transparency due to corneal oedema. To determine the postoperative graft thickness of the donor stroma manual measurements were performed using anterior segment Spectral-Domain Optical Coherence Tomography (SD-OCT) images.

### Statistical analyses

Descriptive data for donor tissue and recipients were collected in a database (Microsoft Excel, version 2208; Microsoft Cooperation, Redmond, WA, USA). The BSCVA was converted to logMAR. Statistical evaluation included paired samples t-test of visual acuity values and calculation of mean and standard deviation using SPSS (version 27.0 IBM SPSS, Armonk, NY, USA).

## Results

Clinical records of 6 consecutive Limbo-DALKs performed in 5 eyes with distinctive corneal pathologies including limbal stem cell deficiency were reviewed.

### Patient characteristics and donor information

Mean age of the patients was 52.0 ± 25.7 years (range 22 to 87), four patients were male and one was female. Causes of limbal stem cell deficiency were thermal and chemical burns, congenital aniridia and chronic inflammatory conditions with Sjögren’s syndrome and recurrent marginal keratitis with necrotizing herpetic keratitis. In three patients the fellow eye was also affected by LSCD (congenital aniridia, Sjögren’s syndrome, ocular chemical burn). A complete ophthalmological examination including best spectacle-corrected visual acuity (BSCVA) and slit lamp biomicroscopy as well as slit lamp photography and corneal Spectral-Domain Optical Coherence Tomography (SD-OCT; Heidelberg Engineering, Heidelberg, Germany) were performed before and after surgery. Preoperative, surgical and postoperative data are shown in Tables 1 and 2.

**Table 1:** Baseline recipient characteristics.

	Age at the time of surgery (years)	Deficiency of limbal stem cells		Preoperative staging	Symblephara	Previous surgeries	Pre-operative stromal thinnest point [μm]	Cause for transplantation	Emergency keratoplasty or HLA-typed	Diameter trephine recipient / donor (mm)	Additional intraoperative measures
		etiology	Extent								
1.1	22	Firework injury leading to perforation and corneal meltdown	total	all 4 quadrants affected stromal > epithelial incl. center	present, nasal lower conjunctiva	3x DALK a chaud, multiple amniotic membrane transplantation	188	Descemetocele with impending perforation	emergency	6.5 / 6.75	+ 2 mg subconjunctival Bevacizumab
1.2	23	2. Limbo-DALK; Patient 1	total	all 4 quadrants affected stromal > epithelial, central cornea containing regular epithelium	present, nasal lower conjunctiva	3x DALK a chaud, multiple amniotic membrane transplantation, 1x Limbo-DALK a chaud	457	Descemetocele with impending perforation	emergency	7,75 / 8.0	
2	46	Chemical burn with nitrogen mustard	Partial (from 3 to 5:30 still partially present)	all 4 quadrants, one quadrant from 3 to 6 o’clock less vascularization Stromal > epithelial	absent	cauterization of corneal neovascularzation, subconjunctival injection of Bevacizumab, EDTA chelation, amniotic membrane transplantation	383	Superficial and stromal opacification	HLA typed	7.5 / 7.75	+ 1 mg subconjunctival Bevacizumab
3	33	Congenital aniridia	total	thin vessels in all 4 quadrants, center involved, stromal	absent	Cataract surgery, Keratectomy of Salzmann nodules Notice: Partner eye: allogeneic limbal stem cell transplantation 10 years earlier (ex domo) followed by rejection 1 year later	No data	Rhegmatogenous retinal detachment and insufficient retinal visualization caused for vitrectomy due to superficial and stromal opacification	emergency	7.5 / 7.75	Macular off retinal detachment: 20Gauge pars plana vitrectomy + explantation of dislocated intraocular lens, retinectomy+ cryotherapy + laser retinopexy + Siluron^®^ 2000 + 1 mg subconjunctival Bevacizumab
4	87	Severe primary Sjögren syndrome	total	all 4 quadrants affected, rather superficial vascularization	present, nasal lower conjunctiva	implantation & explantation of a Krumeichring 10 years before, vascular cautery 6 years before, cataract surgery	473	Superficial and stromal opacification	HLA typed	7.0 / 7.25	+ vascular cautery, lamellar keratectomy + 1 mg subconjunctival Bevacizumab
5	68	Marginal keratitis with corneal ulcer and increasing descemetocele, necrotizing herpes keratitis (HSV1)	partial	at 3 and 9 o’clock no vessels, superficial vessels. Massive growth within last 2 months before Limbo-DALK	absent	3x amniotic membrane transplantation, pannectomy	101 to 342	Descemetocele with impending corneal perforation	emergency	7.0 / 7,25	

**Table 2:** Post-operative course.

	Duration until complete epithelialization	Additional postoperative epithelial defects	Postoperative stromal thinnest point [μm] 1. 1–2 week 2. 6–12 weeks postop	complications	Vascularization of grafted limbus yes/no	Visual acuity [logMAR) • preoperatively • Last-follow up (time point after surgery)	Postoperative endothelial decompensation
**1.1**	Persistent epithelial defect	-	1. 785 2. 514	2 rebubblings, suture loosening, 3 amniotic membrane transplantations, synechiolysis,	yes	• 1.7–2.0 • 1.0 (8 months)	No
**1.2**	1 month	No	1. 506; 768 including donor lamella remnants 2. 497; 636 with pre-limboDALK donor lamella remnants	1 rebubbling	yes	• 2.3 (hand movement) • 1.7 (6 months)	No
**2**	2 weeks	No	1. 473 2. 476	Elevation of liver enzymes with mycophenolate mofetil, thread loosening	yes	• 1.4 • 0.5 (18 months)	No
**3**	2 days	2 months postoperatively (1x1 mm)	1. No data available 2. 688	suture loosening, IOP decompensation, silicone oil associated endothelial decompensation	yes	• 2.3 (hand movement) • 1.5 (18 months)	Incipient endothelial decompensation
**4**	2 days	No	1. No data available 2. No data available - After 6 months: 546	None	yes	• 2.3 (hand movement) • 0.9 (8 months)	No
**5**	2 weeks	3 to 4 months postoperatively (1.2x1 mm) on remnants of band keratopathy	1. 611 2. 579	3 rebubblings 1 EDTA abrasion of a peripheral graft calcification at 10 o’clock	no	• 1.4 • 1.0 (6 months)	No

IOP = Intraocular pressure, CNV = Corneal neovascularization

Mean age of the donors was 69.3 years (range 55 to 90 years), graft diameter varied between 6.75 and 8 mm. Two grafts were HLA typed.

Visual acuity was severely impaired in all cases, resulting in some degree of legal blindness. Three eyes had stage III and three eyes had stage IIB LSCD [16]. Superficial and stromal corneal neovascularisation was present over the complete circumference in all patients. All patients had previous corneal surgery in the affected eye, including repeated amniotic membrane transplantats, cauterisation of corneal vascularisation, abrasion, or previous cataract surgery. One patient had received several emergency DALKs, and one patient had a history of a Krumeichring that had been explanted 10 years previously (see Table 1). Autologous serum eye drops (patient no. 2,3,5), cortisone eye drops (no. 1,5), cyclosporine eye drops (no. 4,5) and artificial tears were also used in all patients to minimise ocular surface inflammation and to preserve the integrity of the ocular surface. None of the corneas of the included patients showed signs of endothelial decompensation before the Limbo-DALK procedure.

In two patients, an HLA-typed corneal transplant was used for Limbo-DALK. In the other four patients, the procedure was performed as an emergency keratoplasty either due to a descemetocele with impending corneal perforation (n = 3) or because of retinal detachment with insufficient visualisation for retinal surgery due to the corneal disease (n = 1). For these patients HLA-typed donor corneas were not available at the time of surgery.

### Corneal epithelisation after surgery

Complete corneal epithelium closure was achieved in two patients (patient no. 3 and 4) after two days and in two patients (patient no. 2 and 5) after 14 days. In one patient (patient no. 1) complete epithelial closure was not achieved after the first Limbo-DALK but after a repeat procedure. After the second procedure, epithelialisation was complete within one month (see Table 2). In one patient (no. 3), small erosion occurred after two months but healed within 2 weeks under conservative treatment.

**Fig 2:**
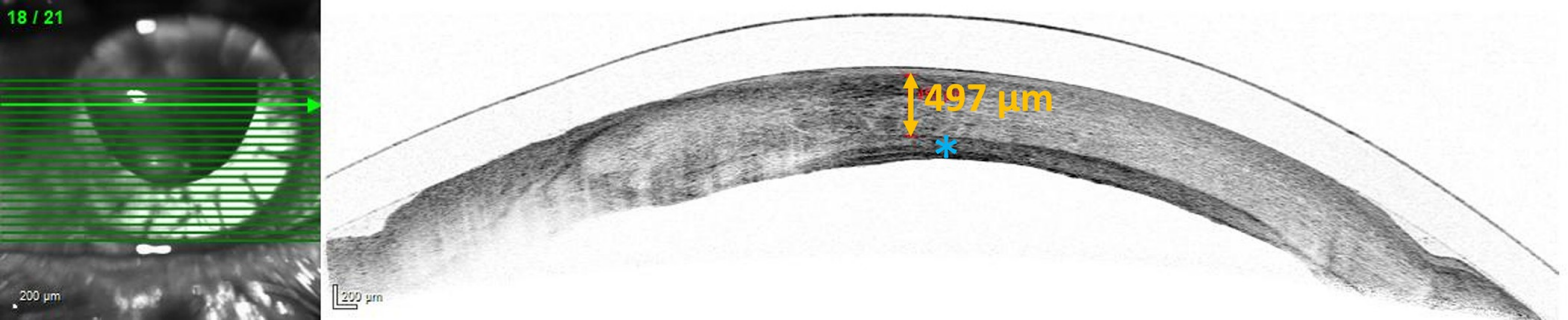
Spectral-Domain Optical Coherence Tomography (Heidelberg Engineering, Heidelberg, Germany) 3 months after the second Limbo-DALK. In addition to the grafted donor lamellae, remnants of deeper corneal stroma (*) can be identified. The thickness of the anterior located full thickness stroma measures 497 μm, indicating healthy recipient’s endothelial cells.

The thickness of the donor stroma was measured using Spectral-Domain Optical Coherence Tomography (SD-OCT) of the anterior segment allowing to exclude corneal oedema. A mean postoperative thickness of 550 μm ± SD 76.8 μm indicated good function of the recipients’ endothelium in all grafts. No corneal edema occurred even after repeat Limbo-DALK (see Fig 2).

**Fig 3:**
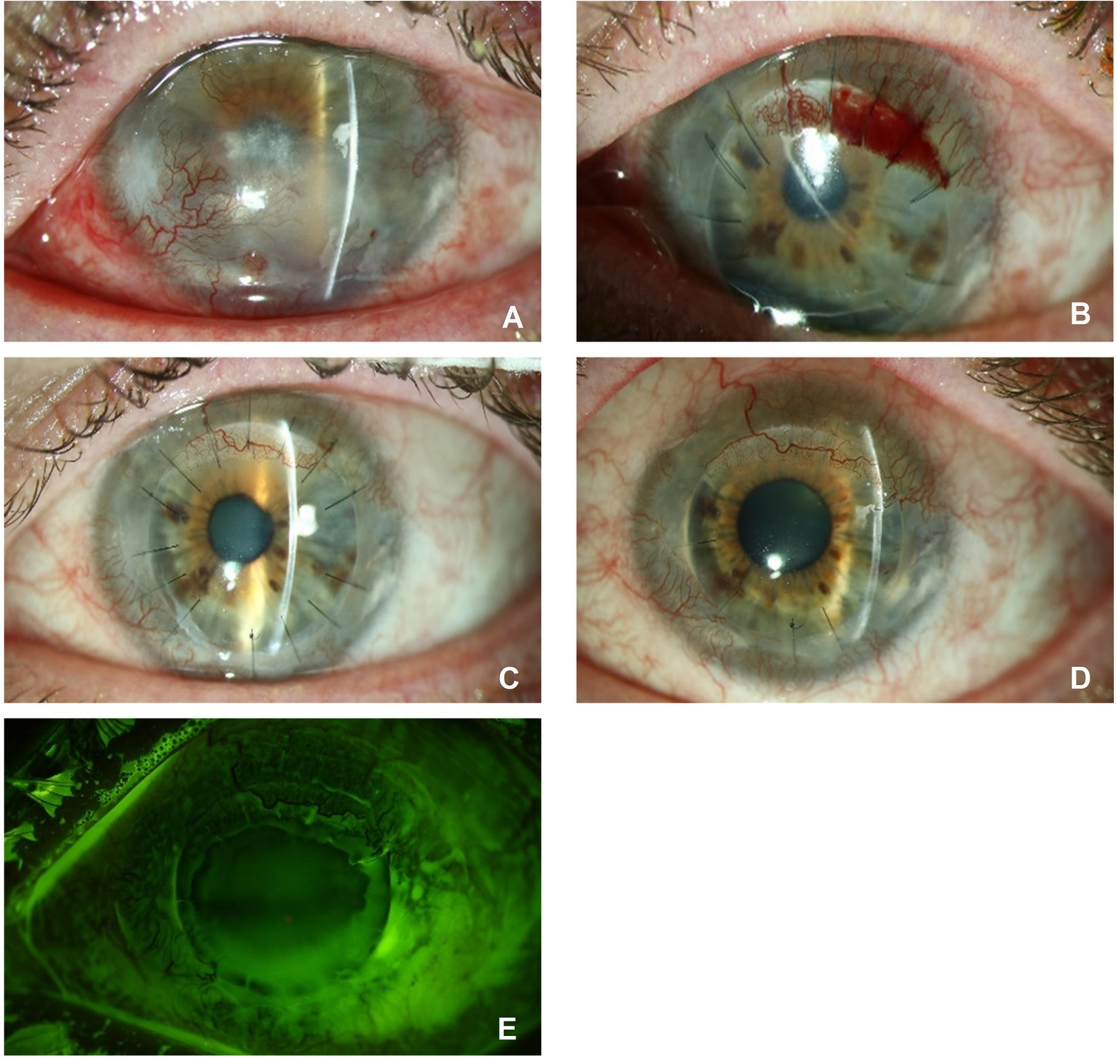
The course of patient 2 at 18 months of follow-up after Limbo-DALK. Preoperatively **(3A)**, limbal stem cell deficiency due to chemical burn caused superficial and stromal corneal neovascularisation in all 4 quadrants, central stromal scarring and lipid keratopathy. Second day after Limbo-DALK **(3B)** with a graft consisting of almost three clock hours of the donor limbus with closed epithelium. A bleeding occurred in the grafted limbus. Six months postoperatively **(3C)**, the limbal bleeding has completely resorbed and a characteristic vascular arcade has formed in the grafted limbus with connection to the recipient vasculature. The epithelium is closed, and the central cornea is clear. 18 months after surgery **(3D**) the graft is still clear with no signs of endothelial decompensation. At this time **(3E)** fluorescein staining indicates complete and smooth epithelialisation of the graft.

Postoperatively, each patient received mycophenolate mofetil 2x 1 g per day per os after surgery. One patient (no. 2, see Fig 3) showed elevated serum liver enzyme during therapy and therefore had to continue treatment with a reduced dosage of 2x 500 mg. One patient (no. 5) with herpetic corneal disease received systemic therapy with acyclovir 400 mg BID until one year after surgery and therefore did not receive systemic immunosuppression.

Only one patient (no. 1) had recurrent epithelial defects and corneal ulceration with relapse of a descemetocele. This patient was therefore treated with a second Limbo-DALK. Despite the high-risk situation no endothelial decompensation occurred within the first 18 months after transplantation. We defined endothelial graft failure as central loss of graft transparency due to oedema. All patients had a clear central corneal stroma without oedema until the last follow-up examination (6 to 18 months, with median follow-up 10.3 months) except for patient (no. 3) who underwent combined retinal and corneal surgery, incipient corneal decompensation due to aphakia and aniridia following intracameral silicone oil was apparent after 18 months. All patients had increased visual acuity after surgery. Pre-operative isual acuity was 1.93 ± 0.44 (mean ± SD) logMAR and improved significantly to 1.10 ± 0.43 logMAR at the last follow-up (p<0.001).

## Discussion

Various surgical techniques for limbal stem cell deficiency have been developed and described over the last 40 years. In 1989, Kenyon et. al. reported human limbal stem cell transplantation via limbal autograft for unilateral limbal stem cell deficiency [17]. Further surgical approaches in the following time comprised a two-step transplantation of an allogeneic full-thickness graft and limbal tissue and supportive treatment with amniotic membrane transplantation and autologous serum eye drops [18, 19]. Around the same time, several group were also attempting to use lamellar dissection of a ring of peripheral cornea, limbus, and adjacent sclera as another variant of allogeneic limbal stem cell transplantation [20–22]. The largest cohort of 43 eyes was reported by the group around Tsubota et al. However, long-term results of most of these procedures were disappointing [20]. Allogeneic central limbo keratoplasty was first introduced by Sundmacher [8]. This procedure combines penetrating keratoplasty with limbal stem cells transplantation in a single procedure. It was hypothesised that maintaining the limbal stem cells in their anatomical niche would lead to favourable results. Rapid visual recovery has been described after these procedures [7, 8, 23].

In general, the long-term prognosis of all allogeneic techniques is limited by immunological reactions against the donor limbal stem cells and the donor corneal endothelial cells [1]. The survival rate of a simple limbal graft is about 50% at 5 years despite systemic immunosuppression, and it is even lower when penetrating keratoplasty (PKP) is performed at the same time [24]. Independent immunological reactions against the grafted limbus and the donor endothelium may occur [9]. We hypothesise, that Limbo-PK has a higher rate of graft rejection compared to Limbo-DALK. The retrospective and single-arm design of our analysis does not allow a direct comparison of the two surgical techniques.

However, in the largest evaluation to date of 192 consecutive allogeneic penetrating Limbo-PK, the group of Reinhard and coworkers identified endothelial graft rejection, in the range of 20% to 45%, as one of the main reasons for graft failure [9].

Compared to PK, DALK offers an increased anterior chamber stability with a reduced risk of intraoperative expulsive choroidal haemorrhage. In 2011, the American Academy of Ophthalmology (AAO) rated DALK as visually equivalent to PK for the treatment of keratoconus, but safer than PK [12]. Previous ocular trauma has been identified as a risk factor for expulsive or pre-expulsive haemorrhages in PK [25]. As many patients with limbal stem cell deficiency also have a history of ocular trauma, patients also benefit from the increased safety of the procedure compared to limbo PK.

One of our patients presented with a retinal detachment. Directly after preparation of the recipient bed, leaving a thin stromal posterior lamella, the anterior chamber was stable, and visualisation was good enough to perform vitrectomy and retinal surgery. This is not usually the case after penetrating keratoplasty.

Perforation of the recipient’s DM can occur intraoperatively necessitating a switch to PK during surgery [11]. In our cohort separation of the recipient’s DM from the stroma was achieved by manual blunt separation with a spatula rather than by deep stromal air injection. Despite manual preparation, recipient’s DM was preserved in all patients.

Human leukocyte antigen (HLA) typing has been suggested by some studies as an important measure to prevent corneal graft rejection after high-risk keratoplasty [1, 6]. As most of our procedures were emergency keratoplasties HLA-matched grafts were used in only two patients. Whether HLA-matching in Limbo-DALKs reduces the risk of immunological reactions against the grafted limbus needs to be answered in larger cohorts.

An alternative approach is the in vitro expansion of stem cells from limbal biopsies, which can be performed as an allogeneic or autologous procedure for unilateral disease [26–28]. However, limbal stem cell transplantation without replacement of the opaque corneal stroma necessitates a second procedure at a later stage to achieve optimal visual acuity. In addition, the cultivation of limbal stem cells takes time, and therefore these cells are not available for emergency interventions. An alternative technique in unilateral LSCD could have been simple limbal epithelial transplantation (SLET), autologous SLET, first described by Sangwan et al. in 2012 in six eyes [29]. In our cohort, there were only two healthy fellow eyes (no. 1 thermal burn, no. 5 necrotizing herpes keratitis). However, both cases required a chaud transplant for descemetocele with impending corneal perforation. Sufficient data on simultaneous SLET with PK or DALK are not available. Keratoplasties were usually performed secondary to SLET [30], but a simultaneous approach or an autologous SLET soon after successful DALK or PK could be an alternative surgical technique to spare systemic immunosuppression in unilateral affected (emergency) patients.

To the best of our knowledge, our study is the first study to describe the new surgical technique of a central Limbo-DALK with segmental limbal transplantation in humans. An experimental study on human donor corneas was conducted by the group of Grueterich et al. [31] They showed in 10 human donor corneoscleral buttons, that the LSK-One microkeratome in combination with the artificial anterior chamber system (ALTK; Moria) allows harvesting of eccentric keratolimbal grafts from donor corneoscleral buttons with 350 μm, 11.0 mm diameter and 3–4 clock hours of limbal area. However, the group also states that the limbal graft should be transplanted in its original position, i.e. at the conjunctival-corneal junction, and not in the midperipheral clear cornea, as recommended by Reinhard and Sundmacher for the Limbo-PKs [1, 7, 8]. According to the stem cell niche described in experimental studies, limbal stroma modulates epithelial differentiation, proliferation and apoptosis in a direction favouring stemness, whereas the corneal stroma promotes differentiation [32, 33]. However, Grueterich et al. also stated that the potential disadvantage of this technique is that we are placing the limbal tissue in a vascularised bed with a higher risk of immune reactions [31]. A recently published case report describes a large-sized ‘deep anterior lamellar limbo-keratoplasty’ for bilateral limbal stem cell deficiency in two cases with LSCD and corneal scarring after chemical injury several years after the accident. The diameter of the donor corneal buttons used was 11.25 and 12 mm diameter in order to capture the limbal stem cells in a circular manner [14]. In our cohort, a central graft of regular diameter was used, allowing the grafted limbus less access to the host vasculature, with a presumed lower risk of rejection.

Our study has several limitations, the most important being the retrospective nature of our analysis. Moreover, each eye of the five patients had a different aetiology of limbal stem cell deficiency, with varying degrees of severity and additional complex anterior or posterior segment disorders, resulting in a highly heterogeneous cohort. In addition, differences in conservative and surgical interventions may influence the results or represent confounding factors. However, preoperative conservative therapy in all patients aimed to reduce surface inflammation while allowing epithelialisation to occur. Intraoperative subconjunctival bevacizumab injection could well be a confounding factor since it showed a negative influence on epithelialisation in laboratory experiments. For this reason, it was not used in the second transplantation in patient no. 1 who failed to epithelialise after the first limbo DALK. The number of patients with LSCD, stromal scarring and intact endothelium is rather rare, but a prospective study with a longer follow-up is needed to confirm the results of this study.

Overall, we conclude that Limbo-DALK is a novel surgical technique and could be a viable alternative to Limbo-PK, allowing corneal stroma and limbal transplantation in patients with LSCD and stromal opacity without the risk of endothelial rejection, even in emergency situations. In a 5-year follow-up of Limbo-PK, graft failure due to endothelial immune reactions/chronic endothelial cell loss also occurred in several patients [1]. In a 20-year follow-up analysis, loss of graft-limbal stem cell function and endothelial graft rejection were identified as the main reasons for graft failure [9]. It is tempting to speculate that in patients with limbal stem cell deficiency and healthy endothelium, the new Limbo-DALK procedure may reduce the overall graft rejection rate and therefore increase graft survival compared to Limbo-PK.

## Supporting information

S1 Dataset (XLSX)
